# Supplementary material for: Systematic assessment of multi-gene predictors of pan-cancer cell line sensitivity to drugs exploiting gene expression data
Source: F1000Res. 2017 Mar 14;5:ISCB Comm J-2927. Originally published 2016 Dec 28. [Version 2] doi: 10.12688/f1000research.10529.2 (PMC5310525; doi:10.12688/f1000research.10529.2)
Supplement: Peer review history

# Peer review: Referee response for version 1

**DOI:** 10.5256/f1000research.11347.r19182

**Published:** 2017-02-13

**Authors:** Ronnie Alves

**Affiliations:** 1 Federal University of Pará , Graduate Program in Computer Science, Belém, Brazil; 2 Instituto Tecnologico Vale, Belém, Brazil

The authors present an empirical modeling approach, highlighting the pros and cons of using a robust machine learning strategy to pan-cancer cell line prediction solely based on gene expression profiles. Single-gene models may not provide an efficient solution on a such high dimensional data, therefore, multi-gene models have been used as a potential alternative to handle the combinatorial space (many candidate gene alterations). Even though the authors introduce quite well the GDSC pharmacotranscriptomic data, I would suggest the addition of more information regarding the baseline / benchmark regarding this classification problem. What would be an acceptable performance prediction? The evolution from single-gene to multi-gene classification could be improved along the feature engineering adopted within these classification strategies. The authors could motivate more the choice of the Random Forest (RF) technique. MANOVA has the problems of handling correlations among dependent variables, and effect size of these correlations. RF provides some improvement on MANOVA's limitations, however, it might suffer from feature subsampling selection and consequently, can overestimate the classification. The author could take a look at this work (Impact of subsampling and pruning on random forests) by Roxane Duroux and Erwan Scornet. ^1^

 Decision tree models are quite tight on training data. Given that the authors used the R language, there are many possibilities of tuning parameters along RF. Importance plots and partial plots could allow to expose features that can help to understand key features of a multi-gene model based on RF. Even though RF has a good performance, one may observe (Figure 4.A and 4.B) that there are some instances where MANOVA is better. The authors could share some light on these observations. Why are those ones hard to classify for RF? Regarding the GDSC data, it is not clear, while splitting the data, whether the data is well balanced along all drugs or not. The authors did well in keeping an independent test data, and it would be interesting to share more information regarding class (127 drugs) distribution along training and test data.

 It would be great of the authors to provide the data and model, so other researchers are able to fully reproduce this study, as well as, devise other robust ensemble learning techniques that might be as good as RF.

 This is an original work and it can be the first, indeed, proposing a benchmark on the estimation of the importance of somatic mutations in drug sensitivity classification.

I have read this submission. I believe that I have an appropriate level of expertise to confirm that it is of an acceptable scientific standard.

## References

[1] DurouxRScornetE: Impact of subsampling and pruning on random forests. *HAL CCSD*.2016;

# Response

**Published:** 2017-03-08

**Authors:** Pedro Ballester

**Affiliations:** INSERM, France

**The authors present an empirical modeling approach, highlighting the pros and cons of using a robust machine learning strategy to pan-cancer cell line prediction solely based on gene expression profiles. Single-gene models may not provide an efficient solution on a such high dimensional data, therefore, multi-gene models have been used as a potential alternative to handle the combinatorial space (many candidate gene alterations). Even though the authors introduce quite well the GDSC pharmacotranscriptomic data, I would suggest the addition of more information regarding the baseline / benchmark regarding this classification problem. What would be an acceptable performance prediction?**

 A drug sensitivity model with MCC>0 on the independent test set can be regarded as an acceptable performance because the performance of classifying cell lines at random is MCC=0. In the context of this study, we are interested in those acceptable models with a MCC value higher than that provided by the best single-gene marker of the same drug (i.e. models with positive MCC in the lower triangular part of Figure 6C). This is now stated in page 8.

**The evolution from single-gene to multi-gene classification could be improved along the feature engineering adopted within these classification strategies. The authors could motivate more the choice of the Random Forest (RF) technique. MANOVA has the problems of handling correlations among dependent variables, and effect size of these correlations. RF provides some improvement on MANOVA's limitations, however, it might suffer from feature subsampling selection and consequently, can overestimate the classification. The author could take a look at this work (Impact of subsampling and pruning on random forests) by Roxane Duroux and Erwan Scornet. Decision tree models are quite tight on training data. Given that the authors used the R language, there are many possibilities of tuning parameters along RF.**

 Thanks for the suggestion. In page 5, we now state that RF is also robust to overfitting, as evidenced in Figure 1. In our experience, going deeper into the tuning control parameters for RF only brings marginal improvements in performance, although it could certainly be interesting from a theoretical point of view.

**Importance plots and partial plots could allow to expose features that can help to understand key features of a multi-gene model based on RF.**

 We agree with the reviewer. However, we think that properly looking at the feature selection/importance question for each of the 127 drugs would require a separated study.

**Even though RF has a good performance, one may observe (Figure 4.A and 4.B) that there are some instances where MANOVA is better. The authors could share some light on these observations. Why are those ones hard to classify for RF?**

 This is certainly an interesting question. For example, Figure 6C shows that 33.9% of the drugs are harder to classify by a multi-variate RF model in the sense that a univariate model performs better. In page 3, we explained that the high dimensionality of the training data sets poses a challenge to classifiers and that these difficulties are drug-dependent. This is due to a number of convoluted factors. First of all, while both models look at the same data in each drug, each model employs a different set of features (genomic vs transcriptomic). Therefore, a single gene mutation might be more predictive of drug sensitivity than a model based on gene expression values in some cases. Second, only a very small subset of features might be predictive of cell line sensitivity to a given drug, which could be challenging for a RF using all the transcriptomic features. Third, the size of training and test sets varies because each drug was tested with a different number of cell lines. Consequently, class imbalances in training set and test set are also different depending on the drug. We are now stating these factors in page 3.

**Regarding the GDSC data, it is not clear, while splitting the data, whether the data is well balanced along all drugs or not. The authors did well in keeping an independent test data, and it would be interesting to share more information regarding class (127 drugs) distribution along training and test data.**

 We completely agree with the reviewer in that it is essential to keep an independent test set to avoid overestimating performance, as standard k-fold cross-validation has been used for both model selection and performance assessment. Full information about the proportion of sensitive and resistant cell lines for each drug can be found in the data sets output by the released software (see below). One can see that training and/or test sets are not well balanced for some drugs and therefore more predictive RF models are likely to be obtained by using strategies to correct for class imbalances. However, the composition of training and test sets should not be altered, as this arise from a time-stamped partition and thus permit a realistic assessment of the performance that can be expected on future data sets (perhaps class imbalanced).

**It would be great of the authors to provide the data and model, so other researchers are able to fully reproduce this study, as well as, devise other robust ensemble learning techniques that might be as good as RF.**

 We have now released the requested R script that was used to facilitate the construction of alternative machine learning models and their validation in the presented benchmark. This is available at http://ballester.marseille.inserm.fr/gdsc.transcriptomicDatav2.tar.gz. We hope that this release will facilitate further improvements on this class of problems.

**This is an original work and it can be the first, indeed, proposing a benchmark on the estimation of the importance of somatic mutations in drug sensitivity classification.**

 We thank the reviewer for his positive assessment of this study. The released software implements this benchmark comprising 127 binary classification problems, one per drug. As drug response data is continuous, it is also possible to use the software to benchmark regression models. Furthermore, the software outputs the results of our study and hence these can be employed as a performance baseline for comparison to the results obtained by the benchmarked models.

# Peer review: Referee response for version 1

**DOI:** 10.5256/f1000research.11347.r20033

**Published:** 2017-02-08

**Authors:** Marc Poirot

**Affiliations:** 1 The Cancer Research Center of Toulouse, INSERM (French National Institute of Health and Medical Research) , Toulouse, France

This is an original research article reporting a machine learning approach exploiting gene expression profiles to predict pan-cancer cell line sensitivity to drugs.

 The title is appropriate and the abstract represent a suitable summary of the work

 I would however suggest that the authors define the abbreviation “GDSC”.

 The paper is well written, the experimental design is good and the conclusions fit with the data.

 Minor point: page 7 figure 3 legend, RC=0.37 is not of the figure: this must be corrected

I have read this submission. I believe that I have an appropriate level of expertise to confirm that it is of an acceptable scientific standard.

# Response

**Published:** 2017-03-08

**Authors:** Pedro Ballester

**Affiliations:** INSERM, France

**This is an original research article reporting a machine learning approach exploiting gene expression profiles to predict pan-cancer cell line sensitivity to drugs.**

**The title is appropriate and the abstract represent a suitable summary of the work**

**I would however suggest that the authors define the abbreviation “GDSC”.**

**The paper is well written, the experimental design is good and the conclusions fit with the data.**

 We thank the reviewer for his positive appraisal of this article. As suggested, we have now defined the abbreviation “GDSC” in the abstract too.

**Minor point: page 7 figure 3 legend, RC=0.37 is not of the figure: this must be corrected**

 Thanks for the observation. RC=0.37 was in mentioned in the part B of the caption, but referred to the part A of the figure. As this is confusing, it has been rewritten to specify that RC=0.37 is the test set recall of the AZ628 (part A) and it compared to that of Sunitinib (RC=0.75) in part B.
